# Lipschütz's ulcers in an adolescent with SARS‐CoV2 infection

**DOI:** 10.1002/ccr3.6503

**Published:** 2022-10-22

**Authors:** Mariana Lira Morais, Mário Moura, Ana Moreira

**Affiliations:** ^1^ Department of Obstetrics and Gynecology Centro Hospitalar Trás‐os‐Montes e Alto Douro Vila Real Portugal

**Keywords:** dermatology, gynecology, pediatrics, Sars‐Cov‐2, vulvar ulceration

## Abstract

Lipschütz's ulcers (LU) are rare entities, which occur mostly in nonsexually active young women. LU appears to be associated with infectious conditions such as Epstein‐Barr virus or cytomegalovirus. We report a case that revealed that SARS‐CoV‐2 infection could be considered a trigger event for the appearance of acute vulvar ulceration.

## Abstract 2

A possible association between Lipschütz's ulcers (LU) and SARS‐CoV‐2 infection had emerged. After excluding other more common causes, this new infectious association should be considered when approaching vulvar ulcer.

## 1 CASE DESCRIPTION

**FIGURE 1:**
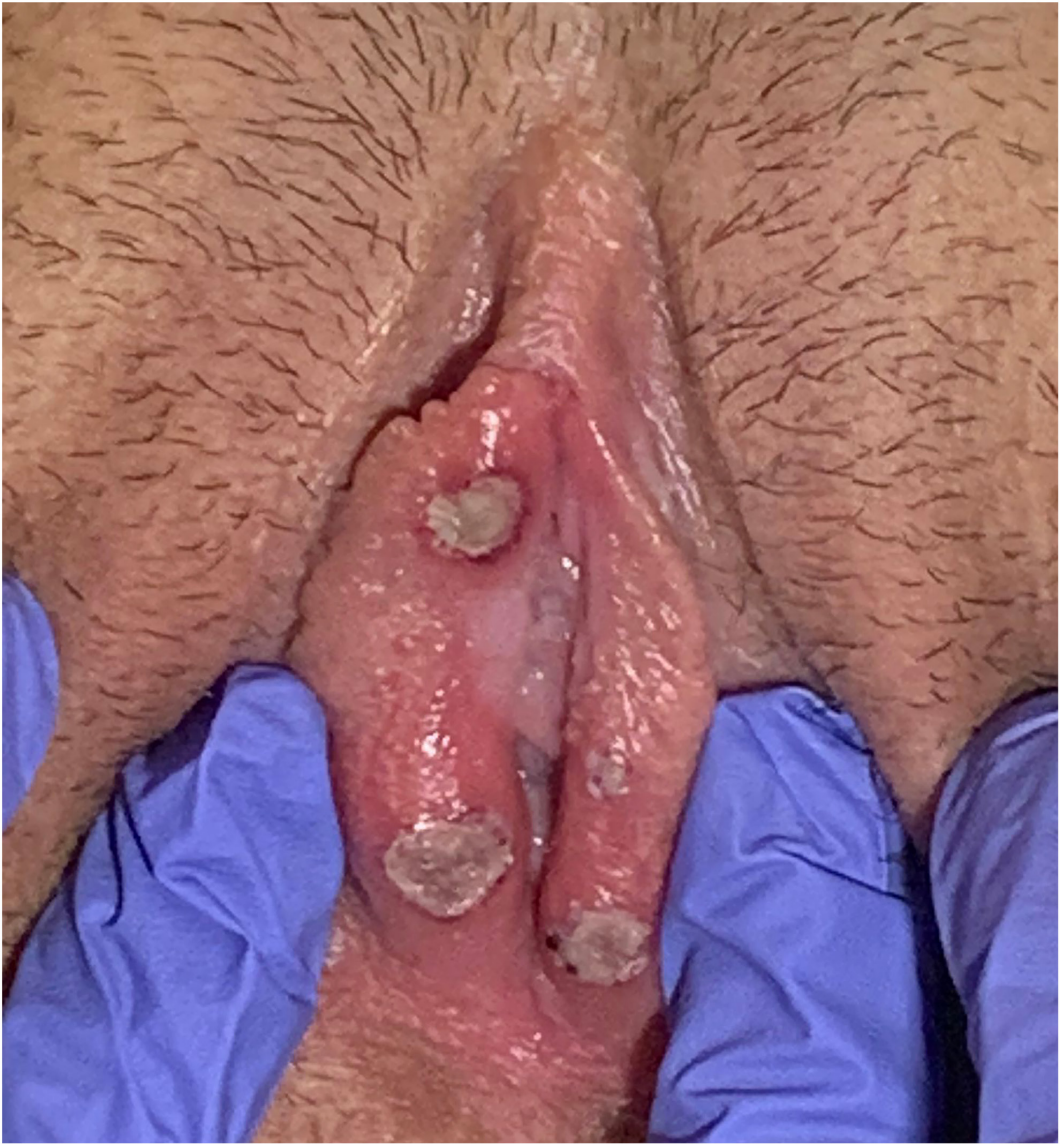
Macroscopic view of acute vulvar ulcerations

We report a Caucasian sexually active 17‐year‐old adolescent who noted flu‐like symptoms and the appearance of multiple, painful vulvar ulcers on the medial side of both *labia minora*, associated with pruritus and dysuria. (Figure 1).

A RT‐PCR SARS‐CoV‐2 test was positive. PCR assay was negative for HSV. Tests for *Chlamydia trachomatis* and *Neisseria gonorrhoeae* and serologic tests for EBV, CMV, syphilis, HIV, and *Mycoplasma pneumonae* were negative. Urinalysis revealed no infection. Behçet's Disease was excluded. The patient was treated with oral anti‐inflammatory drugs and topical lidocaine. 2 weeks later she was asymptomatic and with complete resolution of the vulvar ulceration. SARS‐CoV‐2 infection was considered the trigger event for the appearance of LU.

Lipschütz's ulcers are rare entities characterized by a sudden onset of necrotic and painful genital ulcers. The mechanism responsible for this lesion is still unknown. It is thought that it is associated with an underlying infectious disease and EBV is the most cited etiology.^1^, ^2^

A diagnosis of LU related to SARS‐CoV‐2 is only possible after excluding other more likely causes.^2^ However, a new infectious etiology should be considered when approaching vulvar ulcers. These clinical cases alert to possible conditions associated to SARS‐CoV‐2, leading us to better know this new virus.

## AUTHOR CONTRIBUTIONS

All authors made substantial contribution to the preparation of this manuscript and approved the final version for submission. **MLM** acquired the image, got the patient's consent, did the literature search, and wrote the main aspects of the manuscript. **MM and AM** revised the manuscript, corrected English language, and added some critically important intellectual content.

## FUNDING INFORMATION

No sources of funding were declared for this study.

## CONFLICT OF INTEREST

No conflict of interest was declared by the authors.

## CONSENT

Written informed consent was obtained from the patient to publish this report in accordance with the journal's patient consent policy.

## Data Availability

Data sharing is not applicable to this article as no datasets were generated or analyzed during the current study.
